# Treatment patterns and survival in hepatocellular carcinoma in the United States and Taiwan

**DOI:** 10.1371/journal.pone.0240542

**Published:** 2020-10-14

**Authors:** Yih-Jyh Lin, Chia-Ni Lin, Tannaz Sedghi, Sylvia H. Hsu, Cary P. Gross, Jung-Der Wang, Shi-Yi Wang

**Affiliations:** 1 Division of General and Transplant Surgery, Department of Surgery, National Cheng Kung University Hospital, College of Medicine, National Cheng Kung University, Tainan, Taiwan; 2 Liver Cancer Collaborative Oncology Group, National Cheng Kung University Hospital, Tainan, Taiwan; 3 Department of Public Health, College of Medicine, National Cheng Kung University, Tainan, Taiwan; 4 Cancer Outcomes, Public Policy, and Effectiveness Research (COPPER) Center, Yale Cancer Center and Yale University School of Medicine, New Haven, Connecticut, United States of America; 5 Department of Chronic Disease Epidemiology, Yale University School of Public Health, New Haven, Connecticut, United States of America; 6 Schulich School of Business, York University, Toronto, Ontario, Canada; 7 Section of General Internal Medicine, Department of Internal Medicine, Yale University School of Medicine, New Haven, Connecticut, United States of America; University of Maryland, UNITED STATES

## Abstract

**Background:**

Survival in hepatocellular carcinoma (HCC) is lower in the USA than in Taiwan. Little is known about the extent to which differences in stage at diagnosis and treatment contribute to this difference. We examined treatment patterns and survival in HCC and analyzed factors driving the difference.

**Methods:**

Using a uniform methodology, we identified patients aged 66 years and older with newly diagnosed HCC between 2004 and 2011 in the USA and Taiwan. We compared treatment within 6 months after HCC diagnosis and 2-year stage-specific survival between the two countries.

**Results:**

Compared with patients in Taiwan (n = 32,987), patients in the USA (n = 7,003) were less likely to be diagnosed as stage IA (4% vs 8%) and II (13% vs 22%), or receive cancer-directed treatments (41% vs 58%; all p < .001). Stage-specific 2-year survival rates were lower in the USA than in Taiwan (stage IA: 57% vs 77%; stage IB: 38% vs 63%; stage II: 40% vs 57%, stage III: 14% vs 18%; stage IV: 4% vs 5%, respectively; all p < .001 except p = .018 for stage IV). Differences in age and sex (combined), stage, and receipt of treatment accounted for 3.8%, 17.0%, and 16.8% of the survival difference, respectively, leaving 62.5% unexplained.

**Conclusions:**

Differential stage at diagnosis and treatment were substantially associated with the survival difference, but approximately two-thirds of the difference remained unexplained. Identifying the main drivers of the difference could help improve HCC survival in the USA.

## Introduction

Hepatocellular carcinoma (HCC), the most common primary malignancy of the liver, [1] is one of the leading causes of cancer-related deaths, particularly in countries with high incidence of HCC [2–5]. In the USA, the incidence of HCC has almost tripled since the 1980s, and HCC is the fastest growing cause of cancer-related death [1, 6–8] HCC survival is worse in the USA than in Taiwan [9, 10] From 1991 to 2005, less than 30% of HCC patients covered by Medicare received any treatment for HCC although such treatment was likely to improve survival [11]. The difference in treatment may contribute the difference of HCC survival between the USA and Taiwan. Despite substantial international variation in HCC incidence and mortality [4, 9, 12, 13] little is known about patterns of HCC treatment across countries.

Differences in stage at diagnosis might also impact survival considerably. Patients with early stage HCC who receive surgical resections or transplantation can achieve a 90% of 5-year survival [14]; in contrast, the 1-year survival rate for advanced HCC is only 12% [15]. In the USA, HCC surveillance in clinical practice is underused because no standardized HCC screening/prevention program exists [16]. In contrast, Taiwan launched a nationwide hepatitis B vaccination program in 1984 and free HCC screening projects in high-risk groups in 1994 [17]. Hence, the prevention and early detection programs may lead to a better HCC survival rate in Taiwan. It is unclear whether HCC stage-specific survival in Taiwan differs from that in the USA.

Accordingly, we analyzed patients in similar databases from the USA and Taiwan and applied a uniform methodology to compare treatment patterns and survival between the two countries. We examined the extent to which stage distribution and treatment patterns may explain the survival differences between the USA and Taiwan. Our findings could provide important insights for managing HCC.

## Materials and methods

### Sources of data

We established population-based cohorts from multiple large-scale databases in the USA and Taiwan. We used the Surveillance, Epidemiology, and End Results (SEER)-Medicare linked database to establish the USA cohort. The SEER Program collects and publishes cancer incidence and survival data from population-based cancer registries, which cover approximately 30% of the USA population [18]. The Medicare program is the primary source of healthcare insurance for Americans above the age of 65. All incident cancer patients reported to tumor registries were cross-matched with a master file of Medicare enrollment. For the linkages, 93% of persons age 65 and older in the SEER files were matched to the Medicare enrollment file [19]. Other investigators have described the process for matching persons in the SEER data with their Medicare records [20]. The Yale Human Investigation Committee determined that this study did not directly involve human subjects.

We retrieved data on Taiwanese HCC patients from linking the Taiwan Cancer Registry database [21], Taiwan Mortality Registry, and Taiwan’s National Health Insurance Research Database [22, 23]. Major providers of cancer care in Taiwan with more than 50 beds are obligated to submit data to the Taiwan Cancer Registry database, which covered 98.4% of new cancer cases in 2012 [24]. The National Health Insurance system in Taiwan now covers 99.6% of Taiwan's population, and 93% of the country's hospitals and clinics are contracted with National Health Insurance. Personal identifiers were encrypted to comply with personal electronic data privacy regulations, and all data were analyzed anonymously in the Health and Welfare Data Science Center. This study was approved by the Institute Research Ethical Committee of the National Cheng-Kung University Hospital (IRB number: A-ER-103-203).

### Creation of HCC cohorts

We created the cohort from the USA and Taiwan using the same methodology. The cohorts included patients aged 66 and older with HCC diagnosed between Jan 2004 and Dec 2011 and who had follow-up through the end of 2013. Variables on patient characteristics, primary treatment performed within 6 months after the HCC diagnosis, and survival status were coded in the same ways for both cohorts. We required one-year claims data to assess comorbidities. The two cohorts were not linked, in accordance with data use requirements.

### Outcomes of interest

The primary outcomes were treatment patterns and survival rates. We used five categories to classify treatment: [25] 1) transplantation or resection, including surgical procedures providing chances for potential cure [26], 2) local ablation therapy including radiofrequency ablation or percutaneous ethanol tumor injection [27] 3) trans-arterial chemoembolization (TACE) [28], 4) chemotherapy [29], and 5) no treatment (none of the preceding treatments). The vital statistics reported in each data set included overall survival. We then calculated stage-specific 1-year and 2-year all-cause survival for both cohorts. Based on the age and sex composition in the 2010 USA Census [30], we also calculated age- and sex-standardized stage-specific survival rates for both cohorts.

### Patient demographic characteristics and clinical conditions

Patient demographic characteristics included age at diagnosis, race, gender, and marital status. We used the system of American Joint Committee on Cancer, 6^th^ edition to stage each patient’s HCC at diagnosis. Stage I was further stratified as IA (tumor size ≤2-cm) and IB (otherwise). We coded relevant clinical conditions based on the International Classification of Diseases, 9^th^ Revision (ICD-9) [31]. We used Elixhauser comorbidity for assessing comorbidities, capturing the top 3 diagnoses at clinical visits and the top 5 diagnoses at hospitalizations [32]. Based on one inpatient diagnosis ICD-9 code or two outpatient diagnosis ICD-9 codes occurring between 12 months before and 12 months after HCC diagnosis, we identified risk factors for HCC for each patient, including hepatitis B virus infection, hepatitis C virus infection, alcoholic related liver disease, and liver cirrhosis.

### Statistical analysis

We used chi-square tests to compare patient characteristics between the cohorts. For each cohort, we analyzed treatment patterns, overall and stratified by stage, and conducted survival analyses with the Kaplan-Meier method, stratified by both stage and treatment received. We used a series of models to estimate HCC survival rates in the USA to determine the relative contributions of demographics, stage at diagnosis, and treatment to the difference in HCC survival rates between the USA and Taiwan cohorts. First, we calculated the observed difference in overall 2-year survival between the USA and Taiwan. Second, we calculated the difference in age- and sex-standardized survival, which removes any effect of these demographic factors on survival. Third, we calculated the age- and sex-standardized survival difference assuming that the USA cohort had a stage distribution identical to that in Taiwan. This reduction in the survival difference represented the improvement in survival from screening. Finally, we assumed that the treatment patterns in the USA were identical to those in Taiwan. The resulting reduction in survival difference indicated the improvement attributable to differences in treatment.

## Results

We identified a total of 7,003 patients in the USA and 32,987 patients in Taiwan with HCC. Table 1 summarizes the characteristics of the two cohorts. HCC patients in the USA tended to be older than their counterparts in Taiwan. Males predominated in both cohorts, with a male to female ratio of about 2:1. Patients in the USA, compared to those in Taiwan, were more likely to have advanced disease and larger tumors at diagnosis. Comorbid conditions, chronic hepatitis C infection, alcoholic-related liver disease, and cirrhosis were more common in the USA cohort than the Taiwan cohort (all p-values < .001). There was no significant difference in chronic hepatitis B infection between the two cohorts (p-value = .13).

**Table 1:** Demographic and clinicopathological characteristics of patients with hepatocellular carcinoma between the USA and Taiwan.

	USA (n = 7003)	Taiwan (n = 32987)	
	No. (%)	No. (%)	*p* value
Age Range, y			
66–69	1,521 (22)	8,411 (26)	< .001
70–74	1,885 (27)	9,970 (30)	
75–79	1,688 (24)	7,872 (24)	
80–84	1,204 (17)	4,596 (14)	
85+	7,05 (10)	2,138 (7)	
Race			
White	5,008 (72)	---	
Black	578 (8)	---	
Other	1,417 (20)	32,987 (100)	
Sex			
Female	2,382 (34)	12,203 (37)	< .001
Male	4,621 (66)	20,784 (63)	
Stage			
IA	243 (4)	2,659 (8)	< .001
IB	1,957 (28)	7,541 (23)	
II	929 (13)	7,137 (22)	
III	1,378 (20)	9,382 (28)	
IV	1,009 (14)	2,776 (8)	
Unknown	1,487 (21)	3,492 (11)	
Tumor Size Range			
≤ 2 cm	468 (7)	4,630 (14)	< .001
2–5 cm	1,967 (28)	12,080 (37)	
5–10 cm	1,858 (27)	7,935 (24)	
> 10 cm	937 (13)	3,722 (11)	
Other/Unknown	1,773 (25)	4,620 (14)	
Elixhauser Comorbidity			
None	854 (12)	5,401 (16)	< .001
1 to 2	2,698 (39)	17,523 (53)	
3 or more	3,451 (49)	10,063 (31)	
Hepatitis B			
Yes	673 (10)	3,367 (10)	.13
No	6,330 (90)	29,620 (90)	
Hepatitis C			
Yes	1,946 (28)	5462 (17)	< .001
No	5,057 (72)	27,525 (83)	
Alcoholic-Related Liver Disease			
Yes	1,085 (16)	286 (1)	< .001
No	5,918 (85)	32,701 (99)	
Cirrhosis			
Yes	3783 (54)	6739 (20)	< .001
No	3220 (46)	26248 (80)	

### Treatment patterns

Treatment patterns differed substantially between the USA and Taiwan (Table 2). Patients in the USA were significantly more likely to have received no treatment than those in Taiwan (59% vs. 42%). Similarly, patients in the USA were less likely than patients in Taiwan to have had particular treatments, including surgery (9% vs. 15%), local ablation therapy (8% vs. 11%), and TACE or chemotherapy (25% vs. 32%) (all p-values < .001). Patients with earlier stages of HCC (IA, IB, or II) in the USA were less likely to undergo potentially curative treatments (surgery or local ablation) than those in Taiwan. Across all stages, patients in the USA were also less likely to undergo TACE. In contrast, patients in the USA were more likely to receive chemotherapy.

**Table 2:** Primary treatment for patients with HCC during the first 6 months by cancer stages in USA and Taiwan.

	Transplant or Resection		RFA or PEI		TA(C)E		Chemotherapy		No Treatment	
	No. (%)		No. (%)		No. (%)		No. (%)		No. (%)	
Stage	USA	Taiwan	USA	Taiwan	USA	Taiwan	USA	Taiwan	USA	Taiwan
Overall	656 (9)	49,73 (15)	520 (8)	3,522 (11)	1,548 (22)	10,266 (31)	170 (2)	301 (1)	4,109 (59)	13,925 (42)
IA	36 (15)	482 (18)	66 (27)^‡^	898 (34)^‡^	49 (20)	653 (25)^†^	0 (0)	^†^	92 (38)^‡^	626 (24)^‡^
IB	291 (15)^‡^	1,938 (26)^‡^	242 (12)^‡^	1,313 (17)^‡^	516 (26)^‡^	2,374 (32)^‡^	33 (2)^‡^	23 (0)^‡^	875 (45)^‡^	1,893 (25)^‡^
II	164 (18)	1,356 (19)	112 (12)	897 (13)	330 (36)^†^^‡^	3,022 (42)^‡^	^†^^‡^	16 (0)^‡^	323 (35)^‡^	1,846 (26)^‡^
III	126 (9)	855 (9)	28 (2)	132 (1)	357 (26)^‡^	2,941 (31)^‡^	40 (3)^‡^	144 (2)^‡^	827 (60)^‡^	5,310 (57)^‡^
IV	11 (1)^‡^	66 (2)^‡^	14 (1)	29 (1)	88 (9)^‡^	425 (15)^‡^	52 (5)^‡^	90 (3)^‡^	844 (84)^‡^	2,166 (78)^‡^
Unknown	28 (2)^‡^	276 (8)^‡^	58 (4)^‡^	253 (7)^‡^	208 (14)^‡^	851 (24)^‡^	45 (3)^‡^	28 (1)^‡^	1,148 (77)^‡^	2,084 (60)^‡^

^**†**^: Cells were collapsed due to low counts.

^‡^: Denotes a subset of nation categories whose column proportions differ significantly from each other at the .05 level tested by Bonferroni correction method. Overall, there were significant differences of proportions of each treatment between USA and Taiwan by stage (p < .001).

RFA: Radiofrequency ablation, PEI: Percutaneous ethanol injection, TA(C)E: Transarterial (chemo) embolization.

### Survival outcomes

Patients with HCC in the USA had lower survival rates than those in Taiwan. The 1-year and 2-year overall survival rates were 36.8% and 23.7% in the USA, respectively, compared to 55.9% and 42.3% in Taiwan, respectively (Table 3, all p-values < .001). We also found significantly lower 2-year survival rates in the USA than those in Taiwan across all cancer stages. We observed similar trends in liver disease-specific survival (S1 Table). The results were similar after adjusting for age and sex: patients in the USA had higher age- and sex-standardized all-cause mortality rates than patients in Taiwan across all cancer stages (S2 Table).

**Table 3:** Survival probabilities, overall and stratified by stages, USA and Taiwan.

	1-Year		2-Year	
Stage	USA	Taiwan	USA	Taiwan
**Survival probability, % (Standard error)**				
Overall	36.8 (0.6)	55.9 (0.3)	23.7 (0.5)	42.3 (0.3)
IA	71.2 (2.9)	88.6 (0.6)	57.2 (3.2)	76.9 (0.8)
IB	53.3 (1.1)	77.5 (0.5)	38.1 (1.1)	62.5 (0.6)
II	58.3 (1.6)	75.4 (0.5)	39.6 (1.6)	57.2 (0.6)
III	27.4 (1.2)	29.3 (0.5)	14.2 (0.9)	17.8 (0.4)
IV	9.4 (0.9)	12.0 (0.6)	3.5 (0.6)	5.2 (0.4)
Unknown	23.5 (1.1)	51.1 (0.8)	11.7 (0.8)	36.8 (0.8)

**Fig 1:**
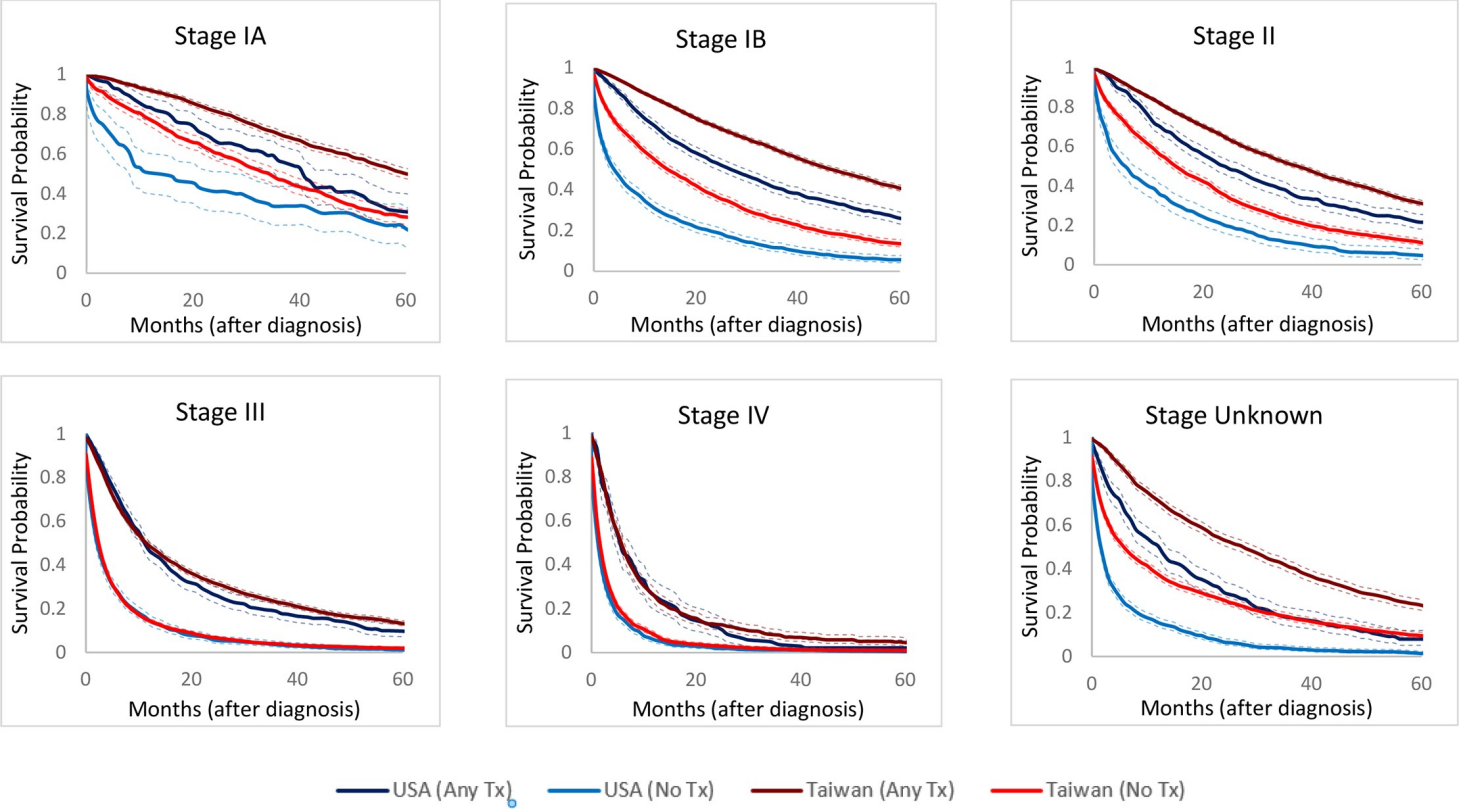
Taiwan, stratified by stage at diagnosis and treatment (Tx) received. **Overall survival in USA vs.** Comparisons of the overall survival for the patients with hepatocellular carcinoma (HCC) stratified by stages and receiving any versus no treatment in USA and Taiwan, including those with unknown stage. Patients with “any treatment” generally had better survival compared with those not receiving treatment. Both of survival rates of patients receiving and not receiving treatment in USA were usually inferior to those in Taiwan for patients of stages IA, IB and stage II HCC. However, there was no statistically significant difference in patients of the stages III and IV HCC.

Fig 1 shows stage-specific survival rates. Compared to patients who did not receive any treatment, patients who received treatment had better survival, both in the USA and Taiwan. The treatment-specific survival rates for US patients with stage IA, IB, II, and stage unknown HCC were worse than that for their Taiwan counterparts. For example, in patients with stage II HCC, the 2-year survival rates in the USA were 50.0% (95% confidence interval [CI] 46.0–53.9%) for treated patients and 20.1% (95% CI 15.9–24.7%) for untreated patients, whereas in Taiwan these rates were 64.8% (95% CI 63.5–66.1%) and 35.4% (95% CI 33.2–37.6%) for treated and untreated patients, respectively. However, there was no significant difference between countries for patients with stage III/IV HCC. In patients with stage IV HCC, the 2-year survival rates in the USA were 11.5% (95% confidence interval [CI] 7.2–16.9%) for treated patients and 1.9% (95% CI 1.1–3.0%) for untreated patients, which were similar to the 12.8% (95% CI 10.3–15.6%) and 3.1 (95% CI 2.4–3.9%) survival rates in treated and untreated patients, respectively, in Taiwan.

### Drivers of the difference in the 2-year survival between the USA and Taiwan

**Fig 2:**
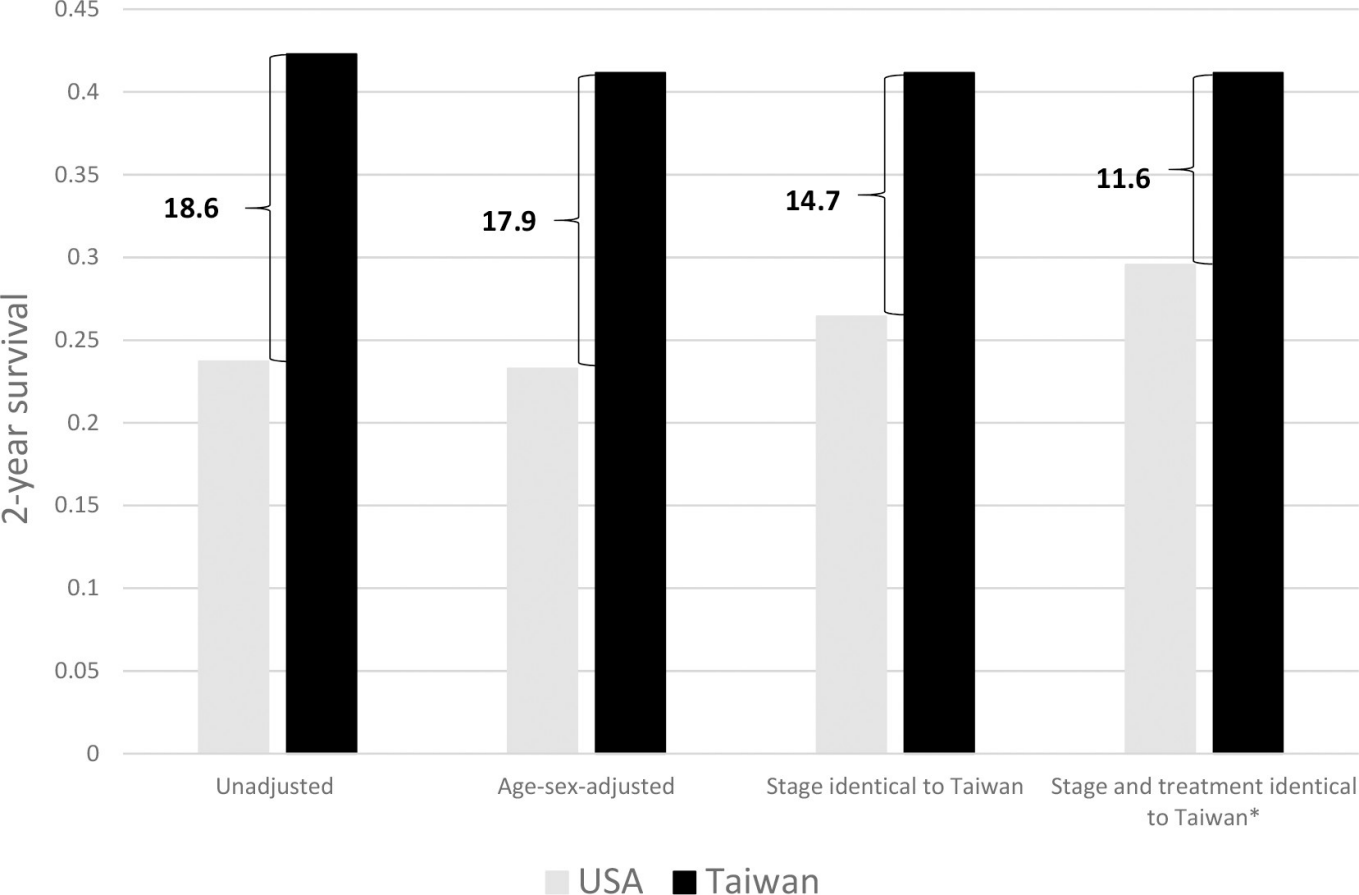
Difference in 2-year survival between USA and Taiwan explained by demographics, stage distribution, and treatment patterns. Comparison of the 2-year survival for the hepatocellular carcinoma (HCC) patients between USA and Taiwan. The age, sex, cancer stage and treatment options were used as parameters for adjustments to delineate the possible mechanism explaining the difference in survival rates between the USA and Taiwan. The unadjusted 2-year survival differed by 18.6% between the USA and Taiwan (the leftmost panel). Age and gender can explain 3.8% of the difference (0.7% divided by 18.6%; the second panel from the left). Stage distributions can further explain 17.0% difference (3.2% divided by 18.6%; the third panel), indicating that early detection through screening is critical. Receiving treatment or not can further explain 16.8%. There is still a 62.5% difference unexplained (11.6% divided by 18.6%; the fourth panel), which deserves further research. *: Assuming the USA had the identical, stage-specific proportions of patients receiving any treatment as Taiwan.

Stage at diagnosis and treatment distributions could explain part of the differences of the survival rates between the USA and Taiwan (Fig 2). The difference in 2-year survival between the USA and Taiwan decreased from 18.6% to 17.9% after age and sex standardization, suggesting that age and sex could explain 3.8% (0.7% divided by 18.6%) of the difference. The survival difference decreased to 14.7% after also adjusting for stage at diagnosis, indicating that early diagnosis in Taiwan could explain an additional 17.0% of the difference. If the proportion of untreated patients in the USA decreased to levels identical to Taiwan, the difference in survival could be further reduced to 11.6%, indicating that receipt of treatment could contribute to a further 16.8% of the difference. Approximately 62.5% of the difference could not be explained after accounting for age, sex, stage at diagnosis, and receipt of treatment.

## Discussion

To our knowledge, our investigation is the first international population-based study of treatment patterns and survival outcomes for patients with HCC. We found that treatment patterns differed substantially between the USA and Taiwan. Notably, patients with HCC in the USA were less likely to receive treatment within six months after diagnosis across different stage subgroups. Using a series of sequential models, we demonstrated that age, sex, stage at diagnosis, and receipt of treatment together explained 33.8% of the difference in 2-year survival between the USA and Taiwan.

Given the absence of universally accepted guidelines for HCC management, variation in treatment strategies between the USA and Taiwan is expected. However, it is concerning that relatively few patients with early-stage HCC in the USA received transplantation or surgical resection, which have potential to cure the disease [14]. In prior research, patients receiving curative treatments (resection and transplantation) had better survival compared to those undergoing ablation or palliative treatments [33–35]. Encouraging curative treatment for eligible, early-stage HCC patients might improve health outcomes substantially [36]. Additionally, patients with advanced HCC in the USA, compared to those in Taiwan, were more likely to receive chemotherapy, but less likely to receive TACE. Therefore, future comparative effectiveness research examining the benefits and harms of different types of HCC treatment would be useful.

Our results build on prior research and have important clinical and policy implications. First, the difference between countries in the proportion of patients receiving any treatment was associated with the difference in survival. In another study, the 5-year survival rate for patients with HCC was 15.2% in the USA and 22.2% in Taiwan without adjusting for receipt of treatment [9]. Our study demonstrated that receipt of treatment explained 16.8% of the difference in the survival rates. As lack of HCC treatment was associated with poor HCC survival in the USA, it is important to identify and address barriers to treatment in the USA.

Second, differences in stage at diagnosis contributed to 17% of the USA-Taiwan survival difference, highlighting the benefits of early diagnosis of HCC. Surveillance of at-risk populations facilitates early cancer detection and could improve outcomes [37, 38]. Although the American Association for the Study of Liver Disease and National Comprehensive Cancer Network recommend a surveillance program with 6-month intervals for patients with liver cirrhosis [39], only 20% of such patients in the USA received regular surveillance prior to a diagnosis of HCC [16, 40, 41]. In contrast, the Taiwanese government has implemented screening programs, and patients with cirrhosis can visit a hepatologist/gastroenterologist without a referral. Our results showed that patients in the USA tended to be diagnosed with larger tumors and at later stages than in Taiwan, highlighting the need for consistent HCC surveillance in the USA [11, 42].

Third, our results showed that factors other than treatment and stage at diagnosis drive 62.5% of the survival difference. Possibilities include other clinical factors, such as etiology of HCC, comorbidities, and liver functions, which could have great impact on HCC survival. Compared to those in Taiwan, HCC patients in the USA were more likely to have hepatitis C, alcoholic related liver disease, and cirrhosis, which not only are risk factors for HCC but also may be associated with poor HCC survival. In a population-based study of French residents, liver cirrhosis did not affect 1-, 3-, and 5-year survival among patients with HCC [43]. Research exploring the role of etiology and cirrhosis on HCC survival difference between the USA and Taiwan is needed. Additionally, survival difference in patients with early-stage HCC in USA vs Taiwan is substantial. Potential explanations include differences in etiology of disease and comorbidity between the two countries, which can have great impact on survival. Furthermore, health care after initial treatments, such as follow-up surveillance to detect HCC recurrence, treatments for recurrence, and supportive care, may differ between the two countries, which may influence survival. For example, co-payment for health care in the USA may be burdensome even though our USA cohort had Medicare coverage. In contrast, patients with cancer in Taiwan can register as suffering from a catastrophic illness and be waived from all co-payments. Hence, post-treatment surveillance and supportive care may be better in Taiwan than in the USA. The continuum of HCC management may also explain the better treatment- and stage-specific survival in early-stage patients in Taiwan than in the USA. Optimal management across the care continuum may improve HCC outcomes in the USA.

Our study has several limitations. Our samples were limited to older individuals and thus our results cannot be generalized to younger patients. Also, samples derived from the SEER-Medicare linked database might not be representative of older patients with HCC in the USA [44], while the cohort in Taiwan’s database are nationally representative of the older population. In addition, both data sets lack information on other factors that impact long-term survival, such as liver function, degree of portal hypertension, α-fetoprotein and other biological markers, and performance status. Patients in the USA were more likely to have comorbidities and liver cirrhosis than those in Taiwan, which may have impacted survival. Furthermore, referral patterns [45] and surgeon specialty [46] might have influenced the HCC therapy patients chose, leading to differences in survival. Finally, because the restrictions of data use agreement, we are not allowed to combine Taiwan’s and USA’s data. We are unable to apply regression models or other statistical methods to directly compare patients in USA vs. Taiwan.

## Conclusions

For older patients with HCC, survival is lower in the USA than in Taiwan. Stage at diagnosis and receipt of treatment differed substantially between the USA and Taiwan. These differences in early diagnosis and treatment contributed to significant survival differences between the two countries. Future research identifying other drivers of the difference in mortality between the two countries could improve HCC survival in the USA.

## Supporting information

S1 Table Liver-disease specific survival, total samples and stratified by stages, USA and Taiwan*. *: Survival using liver disease-specific death was calculated by patients diagnosed between 2004 and 2009 with follow-up until Dec 31st, 2011. ICD-9 of 155, 571 or ICD-10 of C22, K70-K77.
(DOCX)

S2 Table Age-and-sex standardized all-cause survival, total samples and stratified by stages, USA and Taiwan. (DOCX)

## References

[1] Stewart BW, Wild CP. World Cancer Report 2014. International Agency for Research on Cancer; 2014. 39432694

[2] Lu SN, Su WW, Yang SS, et al Secular trends and geographic variations of hepatitis B virus and hepatitis C virus-associated hepatocellular carcinoma in Taiwan. Int J Cancer. 2006;119(8):1946–1952. 10.1002/ijc.22045 16708389

[3] Mittal S, El-Serag HB. Epidemiology of hepatocellular carcinoma: consider the population. J Clin Gastroenterol. 2013;47 Suppl:S2–6. 23632345 10.1097/MCG.0b013e3182872f29 PMC3683119

[4] McGlynn KA, Petrick JL, London WT. Global epidemiology of hepatocellular carcinoma: an emphasis on demographic and regional variability. Clin Liver Dis. 2015;19(2):223–238. 10.1016/j.cld.2015.01.001 25921660 PMC4712629

[5] Ministry of Health and Welfare T. 2016 Statistics of Cause of Death. https://www.mohw.gov.tw/cp-3327-33592-2.html. Accessed 23 Feb, 2018.

[6] El-Serag HB. Hepatocellular carcinoma. N Engl J Med. 2011;365(12):1118–1127. 10.1056/NEJMra1001683 21992124

[7] Altekruse SF, McGlynn KA, Reichman ME. Hepatocellular carcinoma incidence, mortality, and survival trends in the United States from 1975 to 2005. J Clin Oncol. 2009;27(9):1485–1491. 10.1200/JCO.2008.20.7753 19224838 PMC2668555

[8] Ryerson AB, Eheman CR, Altekruse SF, et al Annual Report to the Nation on the Status of Cancer, 1975–2012, featuring the increasing incidence of liver cancer. Cancer. 2016;122(9):1312–1337. 10.1002/cncr.29936 26959385 PMC4840031

[9] Allemani C, Weir HK, Carreira H, et al Global surveillance of cancer survival 1995–2009: analysis of individual data for 25,676,887 patients from 279 population-based registries in 67 countries (CONCORD-2). Lancet. 2015;385(9972):977–1010. 10.1016/S0140-6736(14)62038-9 25467588 PMC4588097

[10] Park JW, Chen M, Colombo M, et al Global patterns of hepatocellular carcinoma management from diagnosis to death: the BRIDGE Study. Liver Int. 2015;35(9):2155–2166. 10.1111/liv.12818 25752327 PMC4691343

[11] Shah SA, Smith JK, Li Y, Ng SC, Carroll JE, Tseng JF. Underutilization of therapy for hepatocellular carcinoma in the medicare population. Cancer. 2011;117(5):1019–1026. 10.1002/cncr.25683 20945363

[12] Parkin DM. International variation. Oncogene. 2004;23(38):6329–6340. 10.1038/sj.onc.1207726 15322508

[13] Global Burden of Disease Liver Cancer C, Akinyemiju T, Abera S, et al The Burden of Primary Liver Cancer and Underlying Etiologies From 1990 to 2015 at the Global, Regional, and National Level: Results From the Global Burden of Disease Study 2015. JAMA Oncol. 2017;3(12):1683–1691. 10.1001/jamaoncol.2017.3055 28983565 PMC5824275

[14] Tadatoshi T, Masatoshi M, Setsuo H, et al Early hepatocellular carcinoma as an entity with a high rate of surgical cure. Hepatology. 1998;28(5):1241–1246. 10.1002/hep.510280511 9794907

[15] Colagrande S, Inghilesi AL, Aburas S, Taliani GG, Nardi C, Marra F. Challenges of advanced hepatocellular carcinoma. World J Gastroenterol. 2016;22(34):7645–7659. 10.3748/wjg.v22.i34.7645 27678348 PMC5016365

[16] Singal AG, Yopp A, C SS, Packer M, Lee WM, Tiro JA. Utilization of hepatocellular carcinoma surveillance among American patients: a systematic review. J Gen Intern Med. 2012;27(7):861–867. 10.1007/s11606-011-1952-x 22215266 PMC3378733

[17] Chen CJ, You SL, Lin LH, Hsu WL, Yang YW. Cancer epidemiology and control in Taiwan: a brief review. Jpn J Clin Oncol. 2002;32 Suppl:S66–81. 11959880 10.1093/jjco/hye138

[18] National Cancer Institute Surveillance Epidemiology and End Results Program. About the SEER Program. 2018; https://seer.cancer.gov/about/. Accessed 17 Feb, 2018.

[19] Sciences NCIDoCCaP. SEER-Medicare: How the SEER & Medicare Data are Linked. 2018; https://healthcaredelivery.cancer.gov/seermedicare/overview/linked.html. Accessed 17 Feb, 2018.

[20] Warren JL, Klabunde CN, Schrag D, Bach PB, Riley GF. Overview of the SEER-Medicare data: content, research applications, and generalizability to the United States elderly population. Medical care. 2002;40(8 Suppl):Iv-3–18. 10.1097/01.MLR.0000020942.47004.03 12187163

[21] Taiwan Cancer Registry. Cancer Statistics. 2018; http://tcr.cph.ntu.edu.tw/main.php?Page=N2. Accessed 3 May, 2018.

[22] National Health Insurance Research Database. Background and data protection,. 2018; https://nhird.nhri.org.tw/en/. Accessed 3 May, 2018.

[23] Ministry of Health and Welfare T. Statistics. 2018; https://dep.mohw.gov.tw/DOS/lp-2499-113.html. Accessed 3 May, 2018.

[24] Chiang CJ, Lo WC, Yang YW, You SL, Chen CJ, Lai MS. Incidence and survival of adult cancer patients in Taiwan, 2002–2012. J Formos Med Assoc. 2016;115(12):1076–1088. 10.1016/j.jfma.2015.10.011 26786251

[25] Forner A, Reig M, Bruix J. Hepatocellular carcinoma. Lancet. 2018;391(10127):1301–1314. 10.1016/S0140-6736(18)30010-2 29307467

[26] Llovet JM, Schwartz M, Mazzaferro V. Resection and liver transplantation for hepatocellular carcinoma. Semin Liver Dis. 2005;25(2):181–200. 10.1055/s-2005-871198 15918147

[27] EASL Clinical Practice Guidelines: Management of hepatocellular carcinoma. J Hepatol. 2018. 10.1016/j.jhep.2018.03.019 29628281

[28] Llovet JM, Bruix J. Systematic review of randomized trials for unresectable hepatocellular carcinoma: Chemoembolization improves survival. Hepatology. 2003;37(2):429–442. 10.1053/jhep.2003.50047 12540794

[29] Thomas MB, Jaffe D, Choti MM, et al Hepatocellular carcinoma: consensus recommendations of the National Cancer Institute Clinical Trials Planning Meeting. J Clin Oncol. 2010;28(25):3994–4005. 10.1200/JCO.2010.28.7805 20679622 PMC2940397

[30] United States Census Bureau. Annual estimates of the resident population by single year of age and sex for the United States: April 1, 2010 to July 1, 2016 (NC-EST2016-AGESEX-RES). Population Estimates, National Population by Characteristics 2010–2016, https://www.census.gov/data/datasets/2016/demo/popest/nation-detail.html. Accessed 2 May, 2018.

[31] Quan H, Sundararajan V, Halfon P, et al Coding algorithms for defining comorbidities in ICD-9-CM and ICD-10 administrative data. Medical care. 2005;43(11):1130–1139. 10.1097/01.mlr.0000182534.19832.83 16224307

[32] Chu YT, Ng YY, Wu SC. Comparison of different comorbidity measures for use with administrative data in predicting short- and long-term mortality. BMC Health Serv Res. 2010;10:140 10.1186/1472-6963-10-140 20507593 PMC2897792

[33] Hoehn RS, Hanseman DJ, Jernigan PL, et al Disparities in care for patients with curable hepatocellular carcinoma. HPB (Oxford). 2015;17(9):747–752. 26278321 10.1111/hpb.12427 PMC4557647

[34] Schwarz RE, Smith DD. Trends in local therapy for hepatocellular carcinoma and survival outcomes in the US population. Am J Surg. 2008;195(6):829–836. 10.1016/j.amjsurg.2007.10.010 18436176

[35] Dong W, Zhang T, Wang ZG, Liu H. Clinical outcome of small hepatocellular carcinoma after different treatments: a meta-analysis. World J Gastroenterol. 2014;20(29):10174–10182. 10.3748/wjg.v20.i29.10174 25110446 PMC4123348

[36] Tan D, Yopp A, Beg MS, Gopal P, Singal AG. Meta-analysis: underutilisation and disparities of treatment among patients with hepatocellular carcinoma in the United States. Aliment Pharmacol Ther. 2013;38(7):703–712. 10.1111/apt.12450 23957569 PMC3777750

[37] Singal AG, Mittal S, Yerokun OA, et al Hepatocellular Carcinoma Screening Associated with Early Tumor Detection and Improved Survival Among Patients with Cirrhosis in the US. Am J Med. 2017;130(9):1099–1106.e1091. 10.1016/j.amjmed.2017.01.021 28213044

[38] Yang JD, Mannalithara A, Piscitello AJ, et al Impact of Surveillance for Hepatocellular Carcinoma on Survival in Patients with Compensated Cirrhosis. Hepatology. 2017. 10.1002/hep.29594 PMC5897179 29023828

[39] Sherman M, Bruix J, Porayko M, Tran T. Screening for hepatocellular carcinoma: the rationale for the American Association for the Study of Liver Diseases recommendations. Hepatology. 2012;56(3):793–796. 10.1002/hep.25869 22689409

[40] Davila JA, Morgan RO, Richardson PA, Du XL, McGlynn KA, El-Serag HB. Use of surveillance for hepatocellular carcinoma among patients with cirrhosis in the United States. Hepatology. 2010;52(1):132–141. 10.1002/hep.23615 20578139 PMC3835698

[41] Mittal S, Kanwal F, Ying J, et al Effectiveness of surveillance for hepatocellular carcinoma in clinical practice: A United States cohort. J Hepatol. 2016;65(6):1148–1154. 10.1016/j.jhep.2016.07.025 27476765 PMC5322857

[42] Singal AG, Yopp AC, Gupta S, et al Failure rates in the hepatocellular carcinoma surveillance process. Cancer Prev Res (Phila). 2012;5(9):1124–1130. 22846843 10.1158/1940-6207.CAPR-12-0046 PMC3435471

[43] Guiu B, Minello A, Cottet V, et al A 30-year, population-based study shows improved management and prognosis of hepatocellular carcinoma. Clin Gastroenterol Hepatol. 2010;8(11):986–991. 10.1016/j.cgh.2010.07.018 20713179

[44] Khalaf N, Ying J, Mittal S, et al Natural History of Untreated Hepatocellular Carcinoma in a US Cohort and the Role of Cancer Surveillance. Clin Gastroenterol Hepatol. 2017;15(2):273–281.e271. 10.1016/j.cgh.2016.07.033 27521507

[45] Hyder O, Dodson RM, Nathan H, et al Referral patterns and treatment choices for patients with hepatocellular carcinoma: a United States population-based study. J Am Coll Surg. 2013;217(5):896–906. 10.1016/j.jamcollsurg.2013.07.007 24041557 PMC4002209

[46] Nathan H, Bridges JF, Schulick RD, et al Understanding surgical decision making in early hepatocellular carcinoma. J Clin Oncol. 2011;29(6):619–625. 10.1200/JCO.2010.30.8650 21205759 PMC4834708

