# Supplementary material for: Treatment patterns and survival in hepatocellular carcinoma in the United States and Taiwan
Source: PLoS One. 2020 Oct 14;15(10):e0240542. doi: 10.1371/journal.pone.0240542 (PMC7556438; doi:10.1371/journal.pone.0240542)
Supplement: S1 Table — *: Survival using liver disease-specific death was calculated by patients diagnosed between 2004 and 2009 with follow-up until Dec 31st, 2011. ICD-9 of 155, 571 or ICD-10 of C22, K70-K77. (DOCX) [file pone.0240542.s001.docx]

**S1 Table.** **Liver-disease specific survival, total samples and stratified by stages, USA and Taiwan†**

|  | **1-Year** | | **2-Year** | | **5-Year** | |
| --- | --- | --- | --- | --- | --- | --- |
| **Stage** | **USA** | **Taiwan** | **USA** | **Taiwan** | **USA** | **Taiwan** |
| **Liver disease-specific survival, % (Standard error)** | | | | | | |
| Overall | 45.5 (0.7) | 59.5 (0.3) | 32.7 (0.7) | 46.5 (0.3) | 18.0 (0.8) | 25.5 (0.4) |
| IA | 77.7 (3.5) | 90.5 (0.7) | 67.8 (4.0) | 79.9 (0.9) | 48.9 (5.2) | 49.8 (1.5) |
| IB | 62.5 (1.4) | 80.6 (0.6) | 49.2 (1.5) | 66.9 (0.7) | 29.3 (1.7) | 41.5 (0.9) |
| II | 67.7 (1.9) | 77.5 (0.6) | 51.7 (2.1) | 60.4 (0.7) | 30.1 (2.5) | 31 (0.8) |
| III | 34.0 (1.6) | 33.3 (0.6) | 21.2 (1.5) | 21.7 (0.5) | 8.9 (1.3) | 9.8 (0.5) |
| IV | 16.2 (1.6) | 16.1 (0.9) | 8.3 (1.3) | 7.9 (0.7) | 3.9 (1.2) | 3.2 (0.5) |
| Unknown | 32.3 (1.5) | 56.7 (1.0) | 18.4 (1.3) | 43.8 (1.0) | 6.2 (1.2) | 20.5 (1.0) |

†: Survival using liver disease-specific death was calculated by patients diagnosed between 2004 and 2009 with follow-up until Dec 31st, 2011. ICD-9 of 155, 571 or ICD-10 of C22, K70-K77
