# Supplementary material for: Treatment patterns and survival in hepatocellular carcinoma in the United States and Taiwan
Source: PLoS One. 2020 Oct 14;15(10):e0240542. doi: 10.1371/journal.pone.0240542 (PMC7556438; doi:10.1371/journal.pone.0240542)
Supplement: S2 Table — (DOCX) [file pone.0240542.s002.docx]

**S2 Table. Age-and-sex standardized all-cause survival, total samples and stratified by stages, USA and Taiwan**

|  | **1-Year** | | **2-Year** | | **5-Year** | |
| --- | --- | --- | --- | --- | --- | --- |
| **Stage** | **USA** | **Taiwan** | **USA** | **Taiwan** | **USA** | **Taiwan** |
| **Survival probability, %** | | | | | | |
| IA | 65.5 | 87.6 | 50.7 | 74.0 | 11.4 | 48.2 |
| IB | 52.0 | 77.2 | 37.4 | 61.1 | 10.2 | 38.5 |
| II | 57.5 | 74.3 | 37.8 | 55.0 | 8.2 | 30.3 |
| III | 27.7 | 29.6 | 13.8 | 17.2 | 2.2 | 8.0 |
| IV | 8.1 | 13.5 | 3.1 | 5.6 | 0.8 | 2.4 |
| Unknown | 26.0 | 52.4 | 13.4 | 37.1 | 1.9 | 17.8 |
